# Increased Expression of Interferon Signaling Genes in the Bone Marrow Microenvironment of Myelodysplastic Syndromes

**DOI:** 10.1371/journal.pone.0120602

**Published:** 2015-03-24

**Authors:** Miyoung Kim, Seungwoo Hwang, Kiejung Park, Seon Young Kim, Young Kyung Lee, Dong Soon Lee

**Affiliations:** 1 Department of Laboratory Medicine, Hallym University Sacred Heart Hospital, Anyang, Republic of Korea; 2 Department of Laboratory Medicine, Seoul National University College of Medicine, Seoul, Republic of Korea; 3 Korean Bioinformation Center, Korea Research Institute of Bioscience and Biotechnology, Daejeon, Republic of Korea; 4 Department of Laboratory Medicine, Hallym University College of Medicine, Anyang, Republic of Korea; 5 Cancer Research Institute, Seoul National University College of Medicine, Seoul, Republic of Korea; University of Texas M.D. Anderson Cancer Center, UNITED STATES

## Abstract

**Introduction:**

The bone marrow (BM) microenvironment plays an important role in the pathogenesis of myelodysplastic syndromes (MDS) through a reciprocal interaction with resident BM hematopoietic cells. We investigated the differences between BM mesenchymal stromal cells (MSCs) in MDS and normal individuals and identified genes involved in such differences.

**Materials and Methods:**

BM-derived MSCs from 7 MDS patients (3 RCMD, 3 RAEB-1, and 1 RAEB-2) and 7 controls were cultured. Global gene expression was analyzed using a microarray.

**Result:**

We found 314 differentially expressed genes (DEGs) in RCMD vs. control, 68 in RAEB vs. control, and 51 in RAEB vs. RCMD. All comparisons were clearly separated from one another by hierarchical clustering. The overall similarity between differential expression signatures from the RCMD vs. control comparison and the RAEB vs. control comparison was highly significant (p = 0), which indicates a common transcriptomic response in these two MDS subtypes. RCMD and RAEB simultaneously showed an up-regulation of interferon alpha/beta signaling and the ISG15 antiviral mechanism, and a significant fraction of the RAEB vs. control DEGs were also putative targets of transcription factors IRF and ICSBP. Pathways that involved RNA polymerases I and III and mitochondrial transcription were down-regulated in RAEB compared to RCMD.

**Conclusion:**

Gene expression in the MDS BM microenvironment was different from that in normal BM and exhibited altered expression according to disease progression. The present study provides genetic evidence that inflammation and immune dysregulation responses that involve the interferon signaling pathway in the BM microenvironment are associated with MDS pathogenesis, which suggests BM MSCs as a possible therapeutic target in MDS.

## Introduction

Myelodysplastic syndromes (MDS) are a group of heterogeneous clonal stem cell disorders characterized by peripheral cytopenia, paradoxical hypercellular bone marrow (BM), and variable degrees of increased blasts [1,2]. The symptoms of MDS are a result of ineffective hematopoiesis, which leads to a peripheral deficiency of maturing blood cells, dysplastic features of hematopoietic cells, dysregulation of the cell cycle, and uncontrolled proliferation. The development of MDS likely occurs through multiple evolutionary stages, and the clinical course is divided into several distinct phases depending on the number of lineages involved in dysplasia and the percentage of blasts in the BM [1,2].

Genetic alterations in hematopoietic cells promote the initiation and progression of MDS. Approximately 40–50% of de novo MDS cases show cytogenetic abnormalities, including partial or complete chromosome loss involving chromosomes 5, 7, 20, 11, and Y [1–3]. These cytogenetic abnormalities are the primary molecular markers or inducers of somatic DNA injury, defective DNA repair, impaired immune surveillance, and dysregulated signal transduction [3,4]. The prognostic importance of these abnormalities is well established, and they are incorporated as key components in the prognostic scoring system for MDS [1,5]. Gene expression studies in MDS cases showed altered expressions of oncogenes (*N-RAS*, *WT1*) and genes involved in cell cycle regulation (*CDKN2B*, *EVI-1*), apoptosis (*BCL2*, *C-MYC*, *TP53*), DNA methylation (*DNMT3A*, *TET2*, *IDH1/IDH2*), and histone deacetylation (*H3K27*, *EZH2*, *UTX*, *ASXL1*) [3–6]. An overexpression of *N-RAS* or *WT1* was observed in advanced stages of MDS, and a down-regulation of *CDKN2B* as a result of promoter hypermethylation was associated with leukemic transformation in MDS [4,7–10].

Nevertheless, previous studies on cytogenetic or molecular genetic alterations of MDS hematopoietic cells do not completely delineate the pathogenesis of this disease, which suggests that the reciprocal interaction between hematopoietic elements and the BM microenvironment might also contribute to MDS pathogenesis [11–15]. Therefore, the role of BM mesenchymal stromal cells (MSCs), which are the main constituents of the BM microenvironment, deserves attention. Previous functional studies primarily focused on disturbances of cytokine production or the supporting ability of the MDS BM microenvironment. The results suggested that the BM microenvironment contributes to the apoptosis of BM hematopoietic cells and the selective growth advantage of CD34+ blasts in MDS [15–17]. Several studies elucidated an altered expression of targeted genes in MDS BM MSCs, but only one published study reported the global gene expression profile of pediatric MDS BM MSCs [18]. However, the pathogenesis and phenotype of pediatric MDS are different from those of adult MDS (inherited BM failure syndrome vs. acquired preleukemic condition, respectively) [1].

We hypothesize that gene expression changes in the BM microenvironment play a fundamental role in the development and progression of MDS. We applied global gene expression profiling to BM MSCs of adult de novo MDS patients and performed pathway analyses to gain insight into the expected consequences of altered gene expression and investigate whether and how the gene expression profile of MDS BM MSCs is distinct from normal individuals. The gene expression profiles of BM MSCs from the RCMD and RAEB subtypes were also directly compared to identify the genes responsible for the progression of the MDS.

## Materials and Methods

### 1. Patients

The study included 7 adults who were referred for lymphoma staging with no evidence of BM involvement as controls and 7 adults who were diagnosed as de novo MDS cases in the Seoul National University Hospital from March 2010 to September 2011. The histopatholgic diagnosis of primary site in control polupation include diffuse large B-cell lymphoma (control01 and control02), extranodal marginal zone B-cell lymphoma of mucosa-associated tissue (control04, control05, control06 and control07), and lymphadenitis (control03). The presence of lymphoma cells in the 7 patients who were referred for lymphoma staging was excluded by thorough (i) morphological examination of BM aspirates and BM biopsy sections and immunohistochemical stains for lymphoid cells (CD3, CD20, CD79a, etc.) as well as (ii) immunoglobulin heavy chain gene and immunoglobulin light chain (kappa) gene rearrangement tests [19,20,21]. All 7 patients showed a normal karyotype in BM, and none of them had a history of treatment for lymphoma. MDS patients included 3 RCMD, 3 RAEB-1, and 1 RAEB-2 (6 males and 1 female, age 41–73 years) according to the WHO 2008 classification [1]. The diagnosis of MDS was made based on the following clinical and laboratory findings: (i) clinical symptoms and signs related to cytopenia; (ii) CBC profile, including WBC differential count; (iii) morphological examination of peripheral blood smear, BM aspiration and biopsy; (iv) BM differential count; and (v) conventional karyotyping. The baseline demographic and laboratory findings are summarized in Table 1. BM aspirates obtained at the time of initial diagnosis were used to establish adherent MSC layers. All participants provided written informed consent to participate in this study. The Institutional Research Board of Seoul National University Hospital approved the study (IRB No. C-1008-012-326).

**Table 1:** Characteristics of MDS patients and normal controls.

Case No. (Sex/Age)	Hb (g/dL)—WBC (002FμL)—PLT (×10^3^/μL)	Diagnosis	Cellularity (%)	Karyotype
RCMD01 (M/41)	5.8–4320–100	MDS, RCMD	90–100	46,XY[20]
RCMD02 (M/65)	8.6–6800–29	MDS, RCMD	90–100	46,XY[20]
RCMD03 (F/69)	9.1–2200–53	MDS, RCMD	80–90	46,XX,9qh-[20]
RAEB01 (M/73)	7.2–8300–108	MDS, RAEB-2	70–80	46,XY,del(20)(q11.2q13.1)[19]/46,XY[1]
RAEB02 (M/73)	8.5–1610–186	MDS, RAEB-1	0–10	45,X,-Y[6]/46,XY,add(3)(q?25),del(5)(q?13),add(16)(q?22)[2]/46,XY[13]
RAEB03 (M/58)	8.0–2650–171	MDS, RAEB-1	70–80	54∼57,XY,+Y,+1,+add(4)(q3?),+add(6)(q13),-8,+9,+9,del(11)(p11.2),-13,add(15)(p10),-17,add(19)(q13.3),+20,+21,+21,+22,+3∼6mar,inc[cp6] /46,XY[14]
RAEB04 (M/58)	10.1–6600–37	MDS, RAEB-1	90–100	47,XY,+8[4]/46,XY[16]
Control01 (M/43)	17.0–5700–290	Normal marrow	40–50	46,XY[20]
Control02 (M/58)	13.3–6990–374	Normal marrow	50–60	46,XY[20]
Control03 (M/34)	13.7–8600–240	Normal marrow	50–60	46,XY[20]
Control04 (F/29)	10.2–5260–246	Normal marrow	20–30	46,XY[20]
Control05 (M/51)	13.8–3840–184	Normal marrow	30–40	46,XY[20]
Control06 (F/48)	10.6–10207–409	Normal marrow	30–40	46,XY[20]
Control07 (M/57)	15.1–6410–171	Normal marrow	50–60	46,XY[20]

### 2. Establishment of adherent MSC layers

Heparinized or EDTA-anticoagulated BM aspirates were used after centrifugation at 2000 rpm for 5 min. The buffy coat layer was mixed with 5 mL of pre-warmed ACK lysis buffer (Lonza, Walkersville, MD, USA), incubated for 5–10 min, and centrifuged at 1200 rpm for 5 min. The supernatant was aspirated, cells were washed with 10 mL of phosphate-buffered saline (PBS), and the mixture was centrifuged at 1200 rpm for 5 min. The supernatant was aspirated, and the cell pellet was mixed with 1.2 mL of pre-warmed MLR media composed of RPMI1640 media (JBI, Seoul, South Korea), 10% FBS (Gibco, Grand Island, NY, USA), 100 units/mL of an antibiotic–antimycotic agent (Gibco), 10 mM HEPES (Gibco), 1 mM sodium pyruvate (Gibco), 4.5 g/L glucose (Gibco), and 0.05 mM 2-mercaptoethanol (Amresco, Solon, OH, USA). The cells were plated onto 20 mm×20 mm glass slides in two 6-well plates (12 wells), and the plates were incubated at 37°C in a 5% CO_2_ incubator until adherent cells (considered BM MSCs) were observed using phase-contrast microscopy. We replaced the culture media every 2–3 days when the confluency of adherent cells reached 30–40%. The cells were harvested at 70–80% confluency. The medium was aspirated, and adherent cells were washed with 2 mL of PBS. The cells were incubated with 500 μL of EDTA-trypsin until the adherent cells detached (∼3–5 min) and mixed with 5 mL of MLR culture media. The mixture was transferred to a 50 mL tube and centrifuged at 1200 rpm for 5 min. The supernatant was removed, the cell pellet was mixed with 10 mL of MLR media, plated onto culture dishes, and incubated under the same conditions (37°C, 5% CO_2_). Cell pellets were mixed with 1 mL of PBS after 3 or 4 passages, counted (usually 1×10^5^–1×10^6^) and stored at −70°C or −140°C in DMSO and 20% FBS.

### 3. Immunophenotypic characterization of MSCs

Surface antigen expression was monitored by flow cytometry using Navios (Beckman Coulter, Brea, CA, USA) and analyzed using Kaluza software (Beckman Coulter). The following antibodies were used: phycoerythrin-cyanin 5 (PC5) mouse anti-human CD34 (581, Beckman Coulter), allophycocyanin (APC) mouse anti-human CD45 (J.33, Beckman Coulter), fluorescein isothiocyanate (FITC, Beckman Coulter) mouse anti-human CD29 (4B4LDC9LDH8, Beckman Coulter), FITC mouse anti-human CD44 (J.173, Beckman Coulter), phycoerythrin (PE) mouse anti-human CD90 (Thy1/310, Beckman Coulter), and PE mouse anti-human CD105 (1G2, Beckman Coulter) [22,23]. More than 5000 labeled cells were acquired and analyzed.

### 4. Gene expression microarray

#### 4.1. RNA preparation

Total RNA was extracted using Trizol (Invitrogen Life Technologies, Carlsbad, CA, USA) and purified using RNeasy columns (Qiagen, Valencia, CA, USA) according to the manufacturers’ protocols. After DNase digestion and clean-up procedures, RNA samples were quantified, aliquoted, and stored at −80°C until used. For quality control, RNA purity and integrity were evaluated using denaturing gel electrophoresis and an OD 260/280 ratio on an Agilent 2100 Bioanalyzer (Agilent Technologies, Palo Alto, CA, USA).

#### 4.2. Labeling and purification

Total RNA was amplified and purified using the Ambion Illumina RNA amplification kit (Ambion, Austin, TX, USA) to yield biotinylated cRNA according to the manufacturer’s instructions. Briefly, 550 ng of total RNA was reverse-transcribed using a T7 oligo(dT) primer. Second-strand gene expression was synthesized, transcribed in vitro, and labeled with biotin-NTPs. After purification, the cRNA was quantified using an ND-1000 Spectrophotometer (NanoDrop, Wilmington, DE, USA).

#### 4.3. Hybridization and data export

Labeled cRNA samples (750 ng) were hybridized to each HumanHT-12 v4 Expression BeadChip for 16–18 h at 58°C according to the manufacturer's instructions (Illumina, San Diego, CA, USA). The array uses 47,231 probes to detect the expression of 34,694 genes. Array signals were detected using Amersham fluorolink streptavidin-Cy3 (GE Healthcare Bio-Sciences, Little Chalfont, UK) following the BeadArray manual. Arrays were scanned with an Illumina BeadArray Reader confocal scanner according to the manufacturer's instructions. Array data export processing and analyses were performed using Illumina BeadStudio v3.1.3 (Gene Expression Module v3.3.8).

#### 4.4. Raw data preparation and statistical analysis

The hybridization quality and overall chip performance were monitored by visual inspection of both internal quality control checks and the raw-scan data. Raw data were extracted using the software provided by the manufacturer (Illumina GenomeStudio v2009.2, Gene Expression Module v1.5.4).

#### 4.5. Microarray data preprocessing

The summarized expression level (AVG_Signal) of each probe was normalized across samples using the quantile method and log2-transformed in the lumi R package [24]. Probe annotation was also retrieved with the lumi R package. Expression data from probes without Entrez Gene ID annotation or correspondence to protein-coding genes were discarded. Expression levels from multiple probes representing the same gene were averaged to yield gene-level expression profiles for 18,581 unique genes.

#### 4.6. Differential expression and clustering analysis

Differentially expressed genes (DEGs) were selected using two criteria: Student’s *t*-test *p*-value < 0.01 and fold change > 1.5. A Venn diagram of DEGs was created in Cytoscape [25] and its MultiColoredNodes plug-in [26]. The overall similarity of differential expression signatures between RCMD vs. control and RAEB vs. control was assessed using the OrderedList R package [27], which does not rely on a simple overlap of DEGs but instead considers the complete ordering of all genes in the two differential expression signatures to assess their overall similarity. Hierarchical clustering was performed in the EMA R package using the average linkage method with a Pearson centered correlation as a similarity metric to examine how well the sample group DEG expression profiles were separable [28]. The expression profile of each gene was standardized prior to clustering using a *z*-transformation such that the row mean and variance were set to 0 and 1, respectively.

#### 4.7. Functional enrichment analysis

Functional enrichment analysis was used to identify prevalent biological themes among the DEGs. Functional enrichment analysis with respect to reactome pathways was performed on the KOBAS web server [29,30] using significance criteria of a gene count ≥ 3 and a *p*-value < 0.01. To simplify the presentation of the results, if several pathways were identified as significant and if they contained the same set of pathway member DEGs, the pathway at the lowest level was selected over pathways at higher levels. For example, interferon signaling was selected over cytokine signaling in the immune system if both pathways contained the same set of member DEGs. Functional enrichment analysis with respect to transcription factor binding sites was performed on the TransFind web server using default options [31].

#### 4.8. Data repository

The microarray data are available at Gene Expression Omnibus under the accession number GSE61853 (http://www.ncbi.nlm.nih.gov/geo/query/acc.cgi?token=chqncqaiztornql&acc=GSE61853 or http://www.ncbi.nlm.nih.gov/geo/query/acc.cgi?acc=GSE61853).

## Results

### 1. Immunophenotypic characterization of MSCs

The majority of the harvested cells from adherent cell layers showed low to intermediate forward scatter and low side scatter in flow cytometric analyses. These cells were positive for CD29, CD44, CD90, and CD105 (known MSC markers) but not CD34 or CD45 (known hematopoietic markers), which is consistent with an MSC immunophenotype (Supplementary Data).

### 2. Differential expression and clustering analysis

**Fig 1:**
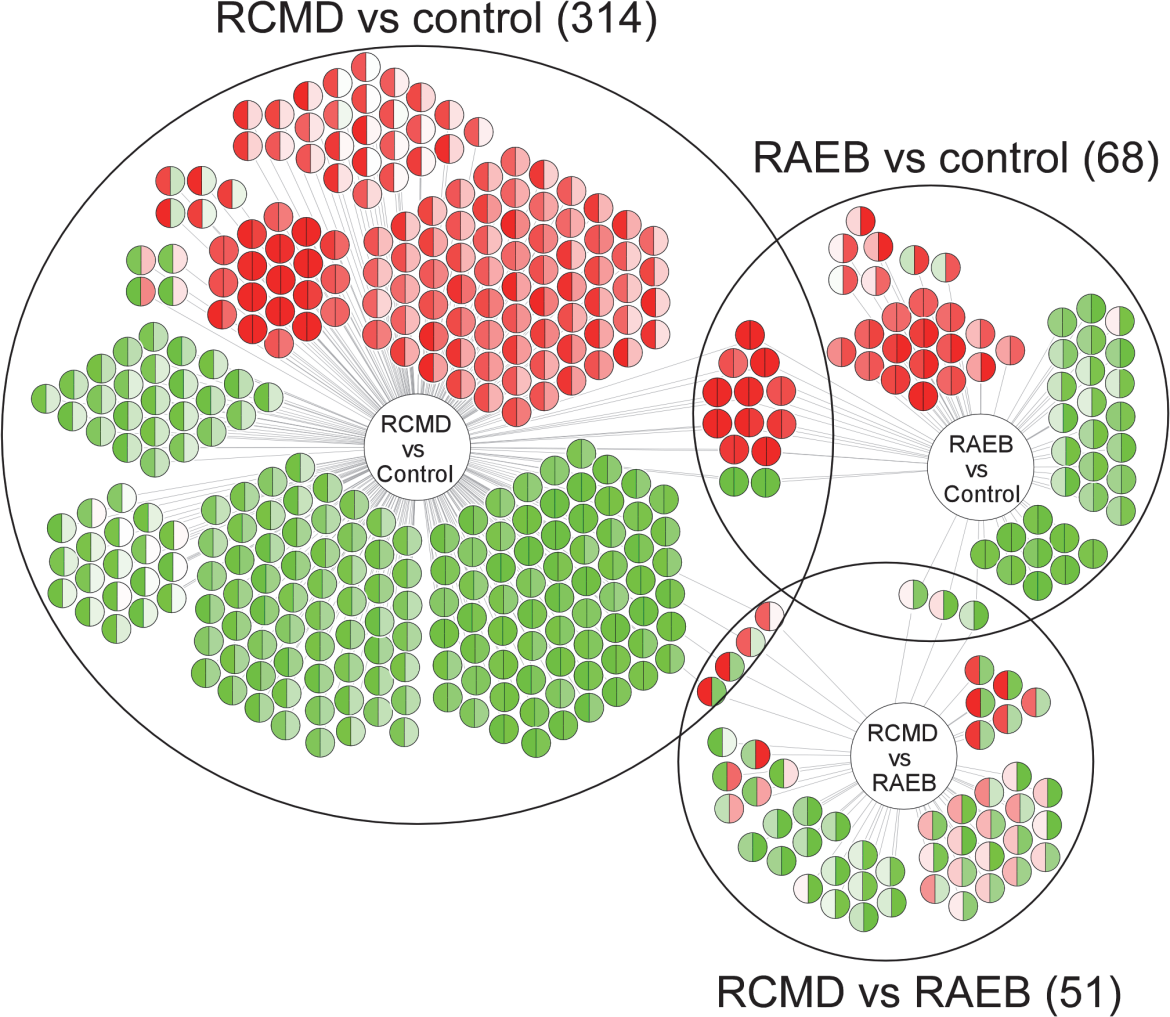
Differentially expressed genes (DEGs) and their overlap in each of the three pairwise comparisons. Each circle represents a DEG. The differential expression in RCMD vs. control and RAEB vs. control is color-coded in the left and right semicircle, respectively. The color intensity is scaled so that more than 2-fold up- and down-regulation correspond to full red and green, respectively.

**Fig 2:**
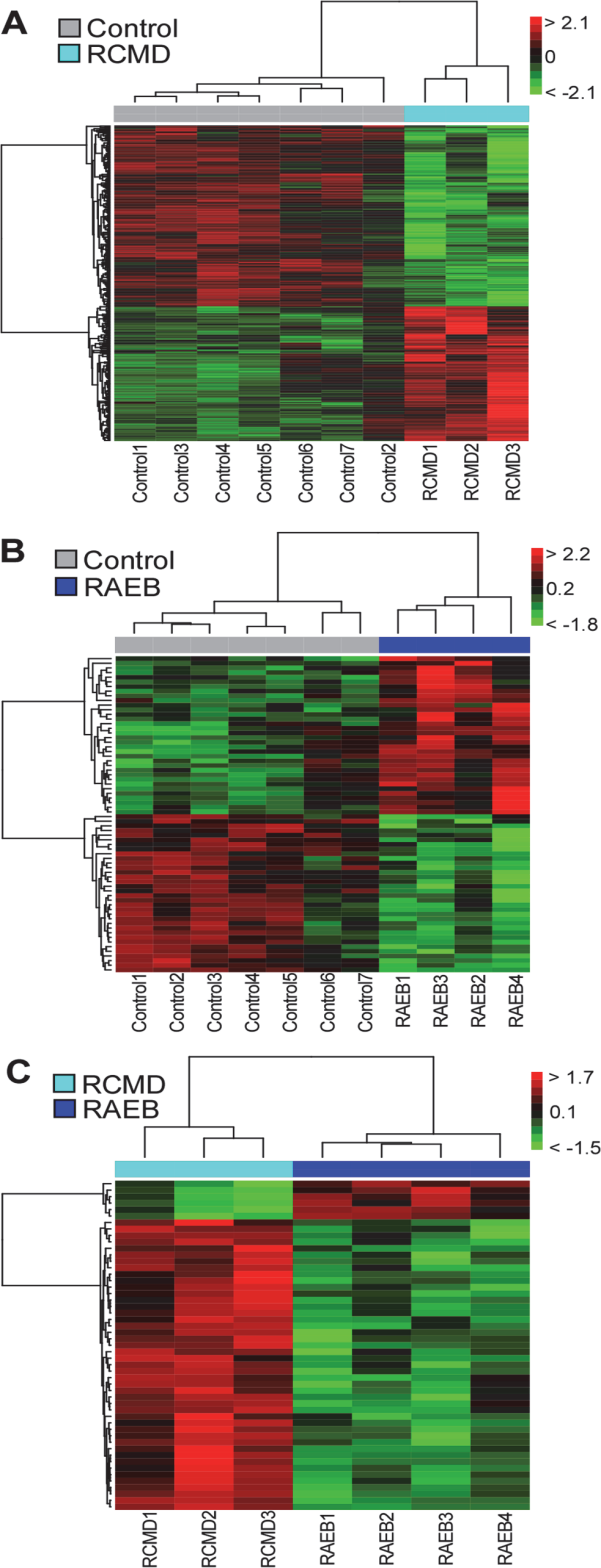
Hierarchical clustering of samples correctly separates all groups: (i) RCMD vs. control, (ii) RAEB vs. control, and (iii) RAEB vs. RCMD. The color intensity is scaled within each row so that the highest expression value corresponds to bright red and the lowest to bright green.

Differential expression was tested for all possible pairwise comparisons between sample groups: (i) RCMD vs. control, (ii) RAEB vs. control, and (iii) RAEB vs. RCMD (Fig. 1). A total of 413 DEGs were identified: 314 in RCMD vs. control (134 overexpressed and 180 underexpressed), 68 in RAEB vs. control (34 overexpressed and 34 underexpressed), and 51 in RAEB vs. RCMD (45 overexpressed and 6 underexpressed). The list of DEGs in each comparison is provided in S1 Data. The number of simultaneous DEGs in at least two comparisons was 3 for RAEB vs. control and RAEB vs. RCMD, 4 for RCMD vs. control and RAEB vs. RCMD, and 13 for RCMD vs. control and RAEB vs. control. The overall similarity between differential expression signatures from the RCMD vs. control and RAEB vs. control comparisons was significant (*p*-value of 0). Hierarchical clustering based on DEG expression profiles clearly separated RCMD from control, RAEB from control, and RCMD from RAEB (Fig. 2).

### 3. Deregulated pathways and transcription factor targets

**Fig 3:**
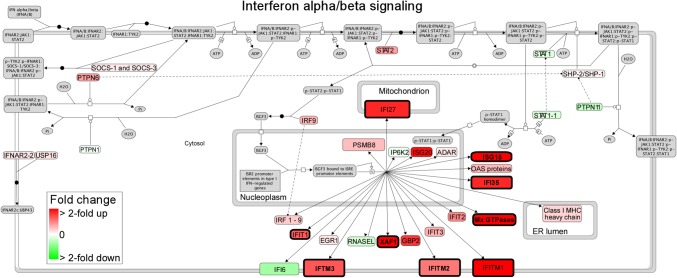
Up-regulation of interferon alpha/beta signaling. Average fold-change values in RCMD vs. control and RAEB vs. control comparisons were used for coloring. Nodes with thick black borders represent differentially expressed genes in the two comparisons.

Compared to control cases, both RCMD and RAEB cases showed a similarly strong up-regulation of interferon signaling pathways, including interferon alpha/beta signaling and the ISG15 antiviral mechanism (Table 2 and Fig. 3). Compared to RCMD, RAEB showed a down-regulation of RNA polymerase I, RNA polymerase III, mitochondrial transcription, GTP hydrolysis, and joining of the 60S ribosomal subunit pathways. Compared to the control, target genes of the transcription factors interferon regulatory factor (IRF) and interferon consensus sequence-binding protein (ICSBP; also IRF8) were up-regulated in RAEB (Table 3). Compared to RCMD, RAEB exhibited a down-regulation of target genes of transcription factor Zic family member 1 (ZIC1), stimulated by retinoic acid 13 (STRA13), upstream stimulatory factor (USF), and aryl hydrocarbon receptor-hypoxia inducible factor (AhR-HIF).

**Table 2:** Significantly enriched reactome pathways in the differentially expressed genes (DEGs).

Reactome pathway	*p*-value	DEG pathway members
Up-regulated in RCMD vs. control		
Interferon alpha/beta signaling	4.66E-10	*IRF7 IFITM3 IFI35 IFITM1 IFITM2 MX2 MX1 IFI6 ISG15*
Interferon signaling	3.05E-09	*IRF7 AAAS IFITM3 IFI35 HLA-DRB4 IFITM1 IFITM2 HLA-DRA MX2 MX1 IFI6 ISG15*
ISG15 antiviral mechanism	1.78E-03	*AAAS MX1 MX2 ISG15*
Immune system	4.05E-03	*IRF7 AAAS MX1 CD74 IFITM3 IFI35 HLA-DRB4 PSMC3 IFITM1 IFITM2 HLA-DRA MX2 CALR UBE2M IFI6 ISG15*
Interferon gamma signaling	9.01E-03	*IRF7 IFITM3 IFI35 ISG15 MX1 IFI6 MX2*
Up-regulated in RAEB vs. control		
Interferon alpha/beta signaling	5.70E-12	*IRF7 IFITM3 IFI35 ISG15 MX1 IFI6 MX2*
ISG15 antiviral mechanism	2.06E-04	*MX1 ISG15 MX2*
Up-regulated in RAEB vs. RCMD		
None		
Down-regulated in RCMD vs. control		
Membrane trafficking	2.64E-05	*YWHAQ AP3S1 YIPF6 VPS4B CLINT1 STAM VAMP2*
Golgi-associated vesicle biogenesis	1.29E-04	*YIPF6 AP3S1 CLINT1 VAMP2*
The citric acid (TCA) cycle and respiratory electron transport	2.27E-03	*ETFDH ETFA NDUFB5 MPC2*
Respiratory electron transport	4.58E-03	*ETFDH ETFA NDUFB5*
Down-regulated in RAEB vs. control		
None		
Down-regulated in RAEB vs. RCMD		
RNA polymerase I, RNA polymerase III, and mitochondrial transcription	1.57E-03	*TAF1B LZTS1 MNAT1*
GTP hydrolysis and joining of the 60S ribosomal subunit	5.55E-03	*EIF1AX EIF3A RPL31*

**Table 3:** Significantly enriched transcription factor binding sites in the promoter sequences of the differentially expressed genes (DEGs).

TF* name	TFBS ID†	FDR^‡^	Target genes in DEGs
Up-regulated in RCMD vs. control			
None			
Up-regulated in RAEB vs. control			
ICSBP (interferon consensus sequence-binding protein, IRF8)	V$ICSBP_Q6	2.18E-04	*UCRP IFI6 IFIT1 IFM3 IN35 MX2 PAR10*
IRF (interferon regulatory factor)	V$IRF_Q6	1.29E-02	*UCRP IFI6 IFIT1 IN35 MX2 PAR10*
Up-regulated in RAEB vs. RCMD			
None			
Down-regulated in RCMD vs. control			
None			
Down-regulated in RAEB vs. control			
None			
Down-regulated in RAEB vs. RCMD			
ZIC1 (Zic family member 1)	V$ZIC1_01	3.87E-03	*B1AJZ9 FHAD1 CE350 PTN7 PDCD4 PLEK2 ACHB4 BAG2*
STRA13 (stimulated by retinoic acid 13)	V$STRA13_01	3.87E-03	*FA21A YAP1 QCR2 ZCH18 TXNL1 MUC24 VATH*
USF (upstream stimulatory factor)	V$USF_01	1.64E-02	*FA21A EIF3 ZCH18 RBX1 MUC24 VATH*
AhR-HIF (aryl hydrocarbon receptor-hypoxia inducible factor)	V$AHRHIF_Q6	1.64E-02	*FA21A TEBP CL023 RGRF1 TXNL1 UGDH*

*TF: Transcription factor

^†^TFBS: transcription factor binding site

^‡^FDR: false discovery rate

## Discussion

The present study determined differences in BM MSC gene expression profiles and the involved pathways between MDS patients and normal individuals. Malignant cells may arise from intrinsic changes, whereas oncogenesis and neoplasm progression may depend on reciprocal autocrine and paracrine communication with the surrounding microenvironment [14,32,33]. The BM microenvironment may play a pivotal role in the pathophysiology of MDS. Several functional studies showed that the BM microenvironment contributes to MDS. Apoptosis of F-36P cells (a MDS cell line) was augmented in the MDS BM microenvironment compared to the normal BM microenvironment [15]. Furthermore, the MDS BM microenvironment inhibited the growth of resident BM hematopoietic cells, likely through an elevated secretion of interferon gamma (IFN-γ), tumor necrosis factor alpha (TNF-α), and interleukin 6 (IL-6) [13,34]. We performed global gene expression profiling of MDS BM MSCs at different disease stages using Illumina BeadArray to improve our understanding of the role of the BM microenvironment in MDS. The combination of pathway analyses and transcription factor binding site analyses facilitated the identification of key pathways and transcription factors that are presumably perturbed in RCMD and RAEB MSCs compared to normal MSCs or between these two different disease stages.

Hierarchical clustering showed that the gene expression profiles in MDS BM MSCs and control BM MSCs were different. Clear separations were observed between MDS BM MSCs and control BM MSCs in two independent pairwise comparisons: RCMD vs. control and RAEB vs. control. Furthermore, similarity analyses revealed that the overall expression signatures of these two MDS subgroups were significantly similar to each other in their differential expression ranks (*p* = 0). Only small overlaps between the two DEG sets were observed despite the strong overall similarity between the differential expression signatures of the two MDS subtypes (Fig. 1). A small overlap problem commonly occurs in gene expression studies because for a gene to be in the intersection of two DEG sets, it must pass the differential expression test two independent times. The probability of simultaneously passing both tests is low because it is equal to the product of the two individual tests’ probabilities, which leads to a small overlap [35]. Nevertheless, Fig. 1 shows that most of the DEGs that were specific to either RCMD or RAEB showed concordant differential expression in the two MDS subtypes, which indicates a common transcriptomic response. Pathway analyses showed that the interferon signaling pathway (interferon alpha/beta signaling and the ISG15 antiviral mechanism) was commonly deregulated in the two comparisons. Similarly, transcription factor binding site analysis revealed that interferon regulatory factor (IRF) and ICSBP (one of 9 IRFs) targeted a significant fraction of the up-regulated DEGs in RAEB. IRFs play diverse roles in innate and adaptive immunity by regulating interferons [36–39]. Cytopenias in MDS may be cytokine induced and associated with T-cell-mediated myelosuppression [40–42]. A derangement of proinflammatory cytokines, including TNF-α, leukemia inhibitory factor, IL-32, IL-1β, IL-6, and hepatocyte growth factor, was observed in the MDS BM microenvironment. These mechanisms suggest that improvements in the impaired erythroid and myeloid colony formation of early hematopoietic progenitors by immune modulating drugs, such as Lenalidomide, may be useful in low-risk MDS [43]. The altered expression of genes associated with interferon signaling of MDS BM MSCs shows that these factors participate in the deregulated inflammatory and immune status of MDS (both RCMD and RAEB), which likely affects and is affected by ineffective hematopoiesis to cause intramedullary apoptosis and an abnormal differentiation/maturation of resident hematopoietic precursors.

We attempted to determine whether similar changes were observed in BM hematopoietic cells in parallel with BM MSCs in MDS patients. However, we could not analyze the gene expression profile of corresponding BM hematopoietic cells. Instead, we compared our data with previously published data (publicly available in Gene Expression Omnibus) of gene expression in MDS CD34+ hematopoietic cells (shown in S1 Data) [44]. The interferon signaling pathway was commonly up-regulated in MDS BM CD34+ cells and BM MSCs, although the altered genes were not identical. The up-regulation of interferon signaling pathways in BM MSCs and BM hematopoietic cells might result from a reciprocal interaction between these cells during the development of the MDS.

Clustering analyses differentiated the gene expression profile of RCMD BM MSCs from RAEB BM MSCs, despite the similar overall differential expression signatures of RCMD vs. control and RAEB vs. control (*p* = 0) and a simultaneous up-regulation of immune (interferon) pathways. These results indicate that gene expression alterations in BM MSCs are involved in disease progression in MDS. The main features of early and late MDS are distinct: ineffective hematopoiesis and bone marrow failure are dominant in RCMD, and a selective growth advantage of blasts is prominent in RAEB [1,7]. Consistent with our hypothesis, direct comparisons of the gene expression profiles of RCMD BM MSCs and RAEB BM MSCs showed that RNA polymerase I, RNA polymerase III, mitochondrial transcription, GTP hydrolysis, and joining of the 60S ribosomal subunit were down-regulated in RAEB BM MSCs. We were unable to directly compare our data with previous data because this study is the first of its type.

The relationship between RNA polymerase and MDS pathogenesis is not well studied. We infer that a deregulation of the RNA polymerase pathway would alter the normal transcription of critical genes, such as tumor suppressor genes, because RNA polymerase plays a role in the modulation of DNA transcription. Recently, mutations in epigenetic regulators (*CDKN2B* and *DNMT3A*) and RNA splicing pathway members (*SF3B1*, *U2AF1*, *ZRSR2*, and *SRSF2*) were recurrently observed in MDS BM hematopoietic cells, and these mutations may be related to the biology and prognosis of MDS [45,46]. Studies on other tumors indicated an association between disease pathogenesis and changes in RNA transcription in tumors and tumor microenvironments. One study on ovarian cancer observed a transcriptional modification resulting from pre-mRNA alternative splicing in cancer cells and the tumor microenvironment [47]. Trabectedin, which is an anti-neoplastic drug for soft tissue sarcoma, interacts with proteins at the site of RNA polymerase II in cancer cells and cells in the tumor microenvironment [48]. Notably, mitochondrial transcription is highlighted in genetic changes in some MDSs (especially RARS). The ringed sideroblasts are erythroblasts that accumulate iron within mitochondria, which indicates a disturbed mitochondrial iron metabolism because of a malfunction in the mitochondrial respiratory chain that may be related to MDS pathogenesis [1,49]. One study of mitochondrial DNA in MDS CD34+ cells using real-time RT-PCR observed a significant reduction in mitochondrial-encoded gene expression in MDS compared to normal controls [50]. A mitochondrial defect has not been studied in the MDS BM microenvironment, and our results demonstrate the possibility that MDS BM MSCs have mitochondrial abnormalities in parallel with resident BM hematopoietic cells.

The down-regulation of DEGs targeted by transcription factors, including ZIC1, STRA13, USF and AhR-HIF, in RAEB BM MSCs compared to RCMD BM MSCs suggests another role of BM MSCs in the progression of MDS. The clinical relevance of this observation is not clear, but we hypothesize that it is associated with features that differ between early and late MDS. The expression of STRA13, USF and AhR-HIF is related to growth arrest and inhibits cell differentiation and proliferation [51–59]. Therefore, RCMD BM MSCs are not competent for differentiation and proliferation, and these cells cannot properly support resident apoptotic hematopoietic cells. In contrast, RAEB BM MSCs may be more supportive for resident cells, including blasts with growth advantages. We suggest that the observations in the pathway analysis and transcription factor binding site analysis in combination with other changes in gene expression analysis lead to complex alterations in MDS BM MSCs rather than resulting in one simple consequence.

One limitation of our study is the small number of samples. Only 7 controls and 7 patients, consisting of 3 RCMD and 4 RAEB patients, were included. A study using a larger sample might provide more reliable information on alterations in gene expression in the MDS BM microenvironment. Nevertheless, our data are likely representative and valid because the gene expression profile was consistent between samples in each group and each subgroup: (i) the gene expression profile was similar among patients but distinguished from the control group; and (ii) the gene expression profile was consistent between patients in each stage (either RCMD or RAEB), but it was different between groups. Above all, our study is noteworthy because there are no published global gene expression profiles of adult MDS BM MSCs. Subsequent studies using a larger study population will provide a better understanding of the relevance of the BM microenvironment in the pathogenesis of MDS.

In summary, gene expression profiles in MDS BM and normal BM microenvironments were distinct. An up-regulation of interferon signaling pathways was exhibited in MDS, which provides evidence for an association of MDS with chronic inflammation or an altered immune system. The down-regulation of several pathways involving RNA polymerase and mitochondria in RAEB compared to RCMD suggests that the BM microenvironment is also involved in MDS progression. Changes in the BM microenvironment according to disease progression are an attractive target for future therapeutic strategies.

## Supporting Information

S1 Data Supporting information. (DOCX)
