# Supplementary material for: Increased Expression of Interferon Signaling Genes in the Bone Marrow Microenvironment of Myelodysplastic Syndromes
Source: PLoS One. 2015 Mar 24;10(3):e0120602. doi: 10.1371/journal.pone.0120602 (PMC4372597; doi:10.1371/journal.pone.0120602)
Supplement: S1 Data — (DOCX) [file pone.0120602.s001.docx]

Supplemenatary figure. Immunophenotypic characterization of adherent cells using flowcytometry. The majority of cells showed low to intermediate forward scatter and low side scatter (Gate S). They were positive for CD29, CD44, CD90, and CD105 (known MSC markers) but not CD34 or CD45 (known hematopoietic markers).

**Figure.**

**
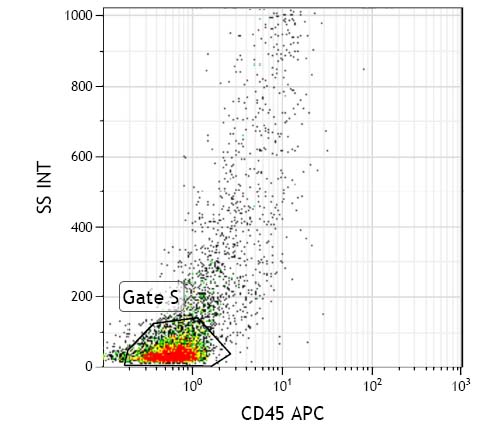

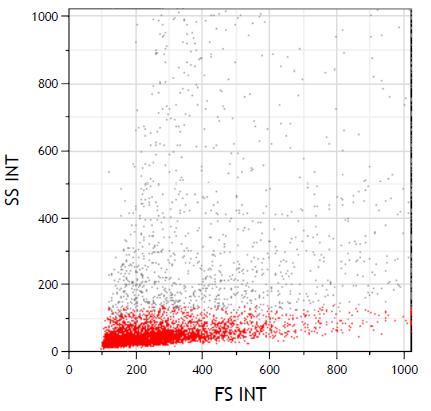
**

**
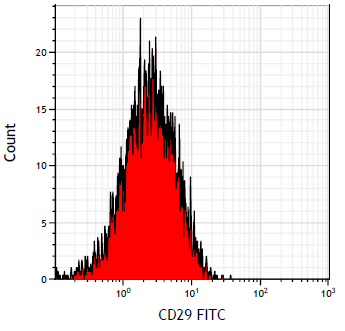

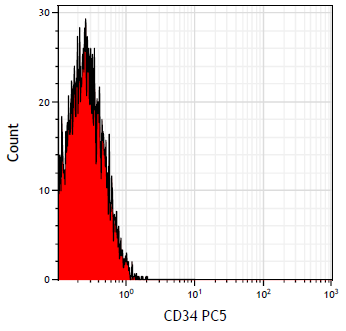
**

**
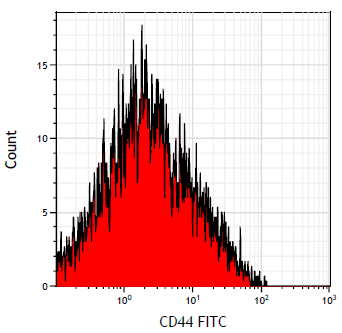

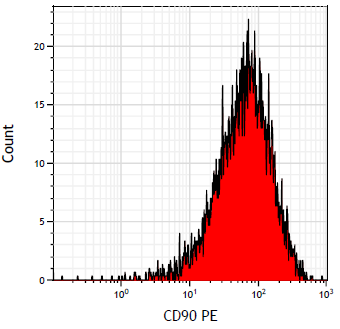

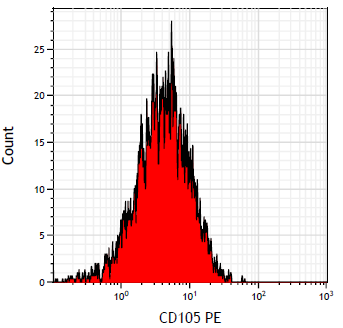
**

**List of differentially expressed genes (DEGs) in pairwise comparisons**

1. DEGs in RCMD vs. control

2. DEGs in RAEB vs. control

3. DEGs in RCMD vs. RAEB

**Comparative analysis with related gene expression dataset of BM CD34+ cells**

Method

We compared our gene expression data with those of Pellagati et al. (Pellagati *et al*, 2010), which is available at Gene Expression Omnibus (GEO) database (accession number GSE19429). Normalized data were obtained and log2-transformed. As in pre-processing of our data, expression data from probes that do not have Entrez Gene ID annotation nor correspond to protein-coding genes were discarded (based on Affymetrix annotation release 34). Then expression levels from multiple probes that represent the same gene were averaged, yielding a gene-level expression profile of 17,729 unique genes. Among them, a total of 17,240 genes were shared with our dataset.

The Pellagati dataset consists of 17 healthy controls and 183 MDS patients, which consists of 55 RA, 48 RARS, 37 RAEB1, and 43 RAEB2. Student’s t-test was performed between all MDS and control samples, early MDS (RA+RARS) and control samples, and RAEB and control samples. For each pairwise comparison, the genes were ranked by t-statistic value to yield a differential expression signature. Then, the ranks of the shared 17,240 genes were compared between differential expression signature of our data and that of Pellagati’s data.

Result

The ranks of genes were summed between the two datasets to identify the most commonly de-regulated genes in the datasets. We listed top five up-regulated genes whose ranks are the most consistent between ours and Pellagati’s, as well as whose Reactome pathway annotation is available (See Table A below). To obtain functional interpretation, functional enrichment analysis was done to top 50 commonly up- and down-regulated genes (Table B). Interferon alpha/beta signaling was shown to be the main biological theme in the commonly up-regulated genes.

**Reference**

Pellagatti, A., Cazzola, M., Giagounidis, A., Perry, J., Malcovati, L., Della Porta, M.G., Jädersten, M., Killick, S., Verma, A., Norbury, C.J., Hellström-Lindberg, E., Wainscoat, J.S. & Boultwood, J. (2010) Deregulated gene expression pathways in myelodysplastic syndrome hematopoietic stem cells. *Leukemia*, **24**, 756-64.

Table A. Top five commonly up-regulated genes in our data and the Pellagati data.

| Symbol | Rank  (Kim) | Rank  (Pellagati) | FC  (Kim) | FC  (Pellagati) | Gene name | Reactome pathway |
| --- | --- | --- | --- | --- | --- | --- |
| Top commonly up-regulated genes in MDS vs. control | | | | | | |
| *IFITM2* | 32 | 84 | 1.53 Up | 1.54 Up | interferon induced transmembrane protein 2 | Interferon alpha/beta signaling |
| *ARAP1* | 17 | 173 | 1.37 Up | 1.22 Up | ArfGAP with RhoGAP domain, ankyrin repeat and PH domain 1 | Rho GTPase cycle |
| *MED16* | 85 | 139 | 1.31 Up | 1.32 Up | mediator complex subunit 16 | Generic Transcription Pathway |
| *IFITM3* | 4 | 276 | 1.66 Up | 1.51 Up | interferon induced transmembrane protein 3 | Interferon alpha/beta signaling |
| *ST3GAL4* | 335 | 50 | 1.32 Up | 1.24 Up | ST3 beta-galactoside alpha-2,3-sialyltransferase 4 | Post-translational protein modification  Metabolism of carbohydrates  Transport to the Golgi and subsequent modification  Signaling by NOTCH |
| Top commonly up-regulated genes in RCMD vs. control and RA+RARS vs. control | | | | | | |
| *MED25* | 15 | 47 | 1.72 Up | 1.30 Up | mediator complex subunit 25 | Generic Transcription Pathway |
| *ST3GAL4* | 26 | 250 | 1.50 Up | 1.19 Up | ST3 beta-galactoside alpha-2,3-sialyltransferase 4 | Post-translational protein modification  Metabolism of carbohydrates  Transport to the Golgi and subsequent modification  Signaling by NOTCH |
| TRAF2 | 152 | 280 | 1.33 Up | 1.33 Up | TNF receptor-associated factor 2 | RIG-I/MDA5 mediated induction of IFN-alpha/beta pathways  TRAF6 mediated NF-kB activation  Apoptosis  TRAF6 mediated IRF7 activation |
| ZNF213 | 13 | 452 | 1.38 Up | 1.17 Up | zinc finger protein 213 | Generic Transcription Pathway |
| SUCLG1 | 96 | 457 | 1.53 Up | 1.11 Up | succinate-CoA ligase, alpha subunit | Citric acid cycle (TCA cycle) |
| Top commonly up-regulated genes in RAEB vs. control | | | | | | |
| *ARAP1* | 2 | 65 | 1.49 Up | 1.26 Up | ArfGAP with RhoGAP domain, ankyrin repeat and PH domain 1 | Rho GTPase cycle |
| *IFITM3* | 49 | 73 | 1.68 Up | 1.66 Up | interferon induced transmembrane protein 3 | Interferon alpha/beta signaling |
| *IFITM2* | 320 | 29 | 1.47 Up | 1.68 Up | interferon induced transmembrane protein 2 | Interferon alpha/beta signaling |
| *CEL* | 190 | 198 | 1.44 Up | 1.29 Up | carboxyl ester lipase | Metabolism of lipids and lipoproteins |
| *ZNF473* | 11 | 392 | 1.12 Up | 1.23 Up | zinc finger protein 473 | Generic Transcription Pathway |

Table B. Significantly enriched Reactome pathways in the commonly de-regulated genes between the two datasets.

| Reactome pathway | P-value | Pathway member genes in DEGs |
| --- | --- | --- |
| Commonly up-regulated in MDS vs. control | | |
| Interferon alpha/beta signaling | 1.51E-05 | *IFITM1 IFITM2 IFITM3 MX2* |
| mRNA processing | 2.22E-10 | *DNAJC8 ZNF473 GTF2H4* |
| Commonly up-regulated in RCMD vs. control and RA+RARS vs. control | | |
| None |  |  |
| Commonly up-regulated in RAEB vs. control | | |
| Interferon alpha/beta signaling | 8.63E-06 | *IFITM2 IFITM3 IFI6 MX2* |
| Signaling by SCF-KIT | 2.58E-03 | *FOXO4 AKT1S1 FES* |
| Commonly down-regulated in MDS vs. control | | |
| None |  |  |
| Commonly down-regulated in RCMD vs. control | | |
| None |  |  |
| Commonly down-regulated in RAEB vs. control | | |
| Apoptosis | 2.11E-03 | *YWHAQ CLSPN PSMA6 HMGB1* |
